# Changing pattern of respiratory virus detections among school‐aged children in a small community – Dane County, Wisconsin, September to December 2022

**DOI:** 10.1111/irv.13171

**Published:** 2023-06-28

**Authors:** Jonathan L. Temte, Maureen Goss, Cristalyne Bell, Shari Barlow, Emily Temte, Allen Bateman, Amra Uzicanin

**Affiliations:** ^1^ University of Wisconsin School of Medicine and Public Health Madison Wisconsin USA; ^2^ Wisconsin State Laboratory of Hygiene Madison Wisconsin USA; ^3^ US Centers for Disease Control and Prevention Atlanta Georgia USA

**Keywords:** acute respiratory illness, influenza, K‐12 schools, pediatric, rhinovirus, SARS‐CoV‐2

## Abstract

Widespread school closures and other non‐pharmaceutical interventions (NPIs), used to limit the spread of SARS‐CoV‐2, significantly disrupted transmission patterns of seasonal respiratory viruses. As NPIs were relaxed, populations were vulnerable to resurgence. This study within a small community assessed acute respiratory illness among kindergarten through grade 12 students as they returned to public schools from September through December 2022 without masking and distancing requirements. The 277 specimens collected demonstrated a shift from rhinovirus to influenza. With continued circulation of SARS‐CoV‐2 and return of seasonal respiratory viruses, understanding evolving transmission patterns will play an important role in reducing disease burden.

## 1 INTRODUCTION

Acute respiratory infections (ARI) in school‐aged children serve as a bellwether for patterns of ARI in the broader community.^1^, ^2^ Prolonged, widespread use of nonpharmaceutical interventions (NPIs) to mitigate the severe acute respiratory syndrome coronavirus 2 (SARS‐CoV‐2) pandemic significantly altered usual circulation of all respiratory viruses.^3^, ^4^ As NPIs were relaxed; however, opportunities for respiratory pathogen resurgence increased. Subsequently, theoretical concerns regarding possibly significant, convergent impacts of respiratory syncytial virus (RSV) and influenza virus, along with SARS‐CoV‐2, became a reality in late 2022.^5^

We used an ongoing community‐based, laboratory‐supported study of ARI in school‐aged children in a small community to characterize occurrences of respiratory viruses in the late SARS‐CoV‐2 pandemic period (September to December 2022), as children returned to public schools in the absence of masking and distancing.

## 2 METHODS

The ORegon CHild Absenteeism due to Respiratory Disease Study [ORCHARDS; Oregon School District (OSD), Oregon, WI; enrollment ~4100 in seven schools)] is a prospective, community‐based observational study enrolling 4‐year‐old kindergarten through 12th grade (4 K‐12; 4‐ to 18‐year‐old) children with ARI after parental consent. ORCHARDS has afforded continuous collection of data and respiratory specimens since January 2015; study design and methods are described in detail elsewhere (Protocol 2013‐1357 approved by University of Wisconsin Health Sciences‐IRB).^6^ Since March 2020, ORCHARDS has utilized participant‐collected respiratory specimens. Specimens are evaluated for SARS‐CoV‐2 and influenza A and B using reverse transcription polymerase chain reaction and for 14 additional viruses using a multiplexed respiratory pathogen panel at the Wisconsin State Laboratory of Hygiene.^6^

The 2022–2023 academic year began on September 1, with winter break commencing on December 22. The OSD did not employ masking or distancing requirements during this observation period. Distancing was discontinued when schools resumed in September 2021, and masking was no longer required starting March 2022. We present the number of detections of respiratory viruses and the percent of detections by week over 16 weeks, from September 5 through December 25, 2022. Absenteeism is expressed as the average daily number of students absent due to illness for each week. We used the Pearson correlation coefficient to compare the number of ORCHARDS participants and the number of illness‐related absences for each week.

## 3 RESULTS

**FIGURE 1:**
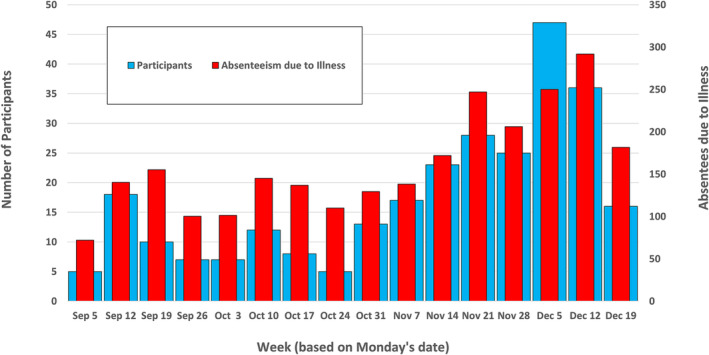
Number of 4 K through 12th grade participants providing respiratory specimens per week (narrow red bars) and average daily illness‐associated absenteeism each week from the Oregon School District (Oregon, WI: wide blue bars) for period from September 5 through December 25, 2022.

**FIGURE 2:**
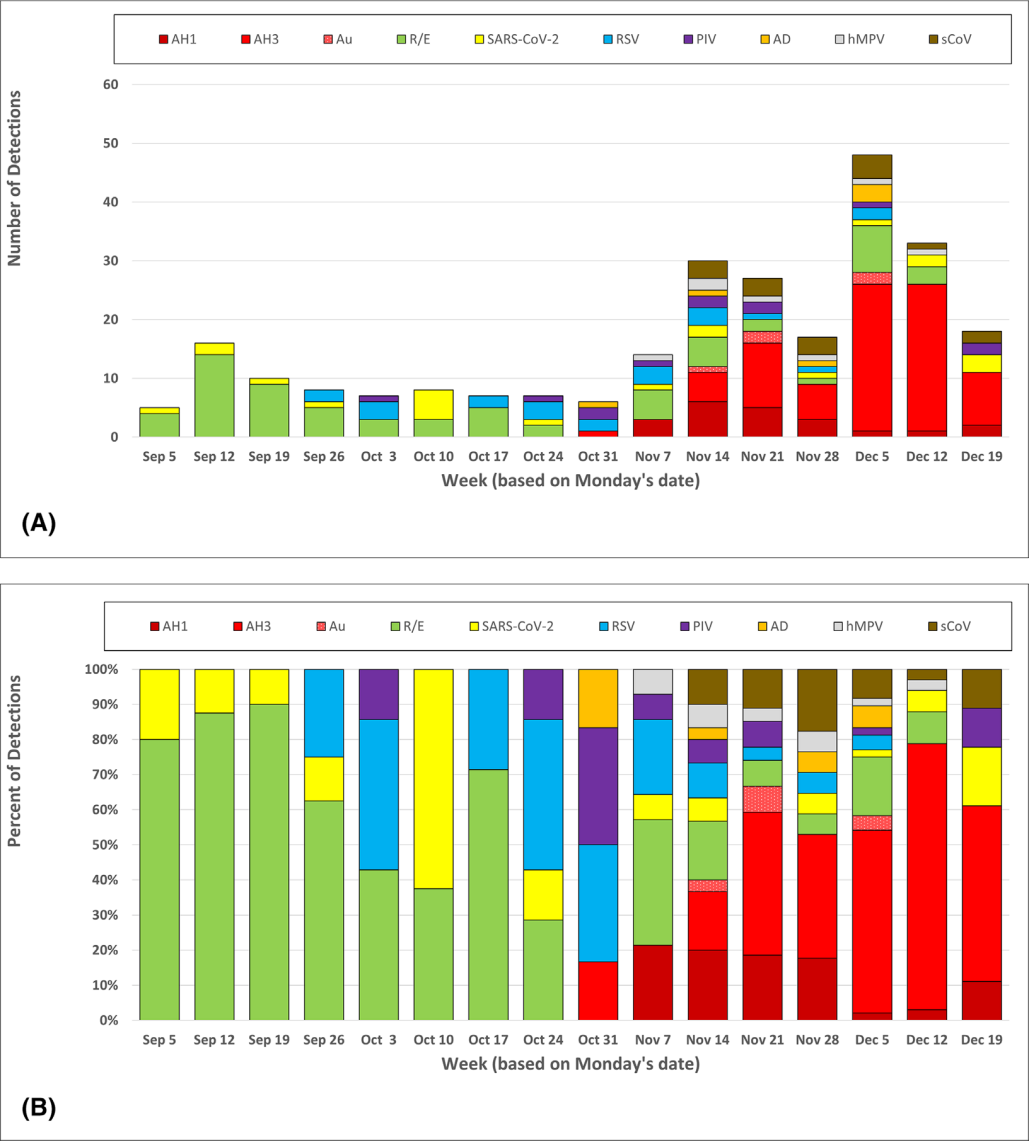
(A) Number of detections of respiratory viruses per week from participants in the ORegon CHild Absenteeism due to Respiratory Disease Study (ORCHARDS, Oregon, WI) from September 5 through December 25, 2022 (AH1 = influenza A(H1); AH3 = influenza A(H3); Au = influenza A unsubtypable; R/E = rhinovirus/enterovirus; SARS‐CoV‐2 = severe acute respiratory syndrome coronavirus 2; RSV = respiratory syncytial virus type A and B; PIV = parainfluenza virus type 1, 2 and 4; Ad = adenovirus; hMPV = human metapneumovirus; sCoV = seasonal coronavirus HKU1 and OC43). (B) Percent of detections by virus type and week.

During the 16‐week period, we received 277 participant specimens. Of these, 229 (82.7%) had positive virus detection, including 28 dual virus detections (10.1%) and two triple virus detections (0.7%). Participants per week mirrored OSD illness‐related absenteeism (Figure 1) and was highly correlated with it (*r* = 0.902; *p* < 0.001). SARS‐CoV‐2 detections were relatively stable over the observation period (mean ± SD: 1.3 ± 1.3 detections/week; Figure 2A and Figure 2B). Rhinovirus/enterovirus (R/E) cases predominated in the first 8 weeks (*n* = 45; 66.2% of detections) declining to 12.4% (*n* = 24) in the second 8 weeks; influenza A was initially detected on November 1 and comprised 56.0% (*n* = 108) of detections in the second 8 weeks, the majority (75.9%; *n* = 82) being H3 viruses.

**FIGURE 3:**
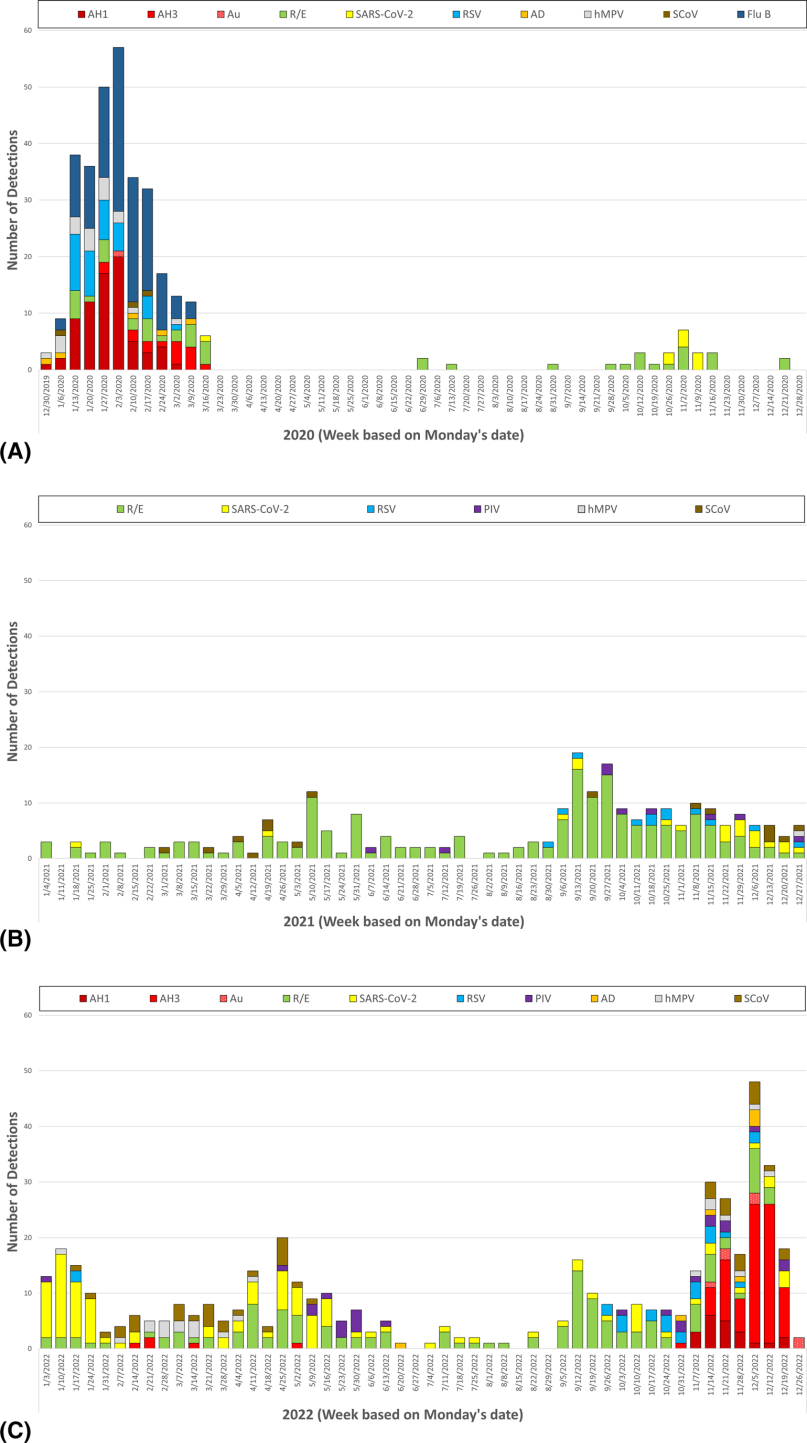
(A) Weekly virus detections for 2020 (AH1 = influenza A(H1); AH3 = influenza A(H3); Au = influenza A unsubtypable; R/E = rhinovirus/enterovirus; SARS‐CoV‐2 = severe acute respiratory syndrome coronavirus 2; RSV = respiratory syncytial virus type A and B; PIV = parainfluenza virus type 1, 2 and 4; Ad = adenovirus; hMPV = human metapneumovirus; sCoV = seasonal coronavirus HKU1 and OC43; Flu B = influenza B). (B) Weekly virus detection for 2021. (C) Weekly virus detection for 2022.

A wide variety of other respiratory viruses were detected including RSVA (*n* = 18), coronavirus‐HKU1 (CoV‐HKU1: 14), human metapneumovirus (hMPV: 7), parainfluenza 4 (PIV4: 7), adenovirus (Ad: 6), RSVB (4), PIV1 (3), CoV‐OC43 (2), and PIV3 (2). The number and diversity of viruses detected during the observation period has not been seen since before the SARS‐CoV‐2 pandemic (Figure 3A–C).

## 4 DISCUSSION

During autumn 2022, as children returned to school without NPIs, a wide variety of respiratory viruses was detected in a small community setting, along with a steady, but low level of SARS‐CoV‐2. Initial dominance of R/E subsided as influenza A virus activity increased in this community. As was seen elsewhere, the influenza season peaked early compared to previous trends.^6^ RSV activity was noted throughout the observational period, but declined as influenza virus increased.

This study's generalizability is limited by the small geographical study area and the possibility of selection bias by participants' willingness to enroll. Conversely, the use of a long‐standing laboratory‐supported, community‐based, participatory platform^4^, ^7^ and previous findings that illness absenteeism mirrors detections of respiratory viruses in this study setting^2^ reinforce the present findings.

As SARS‐CoV‐2 transitions into endemicity and NPI use subsides, other respiratory viruses are returning to prominence in communities calling for better understanding of respiratory virus patterns.

## AUTHOR CONTRIBUTIONS

**Jonathan L. Temte:** Conceptualization; formal analysis; funding acquisition; investigation; methodology; visualization; writing‐original draft; writing‐review and editing. **Maureen Goss:** Data curation; methodology; project administration; validation; writing‐original draft. **Cristalyne Bell:** Data curation; formal analysis; writing‐original draft; writing‐review and editing. **Shari Barlow:** Funding acquisition; methodology; project administration; supervision; writing‐review and editing. **Emily Temte:** Data curation; methodology; project administration; validation; writing‐review and editing. **Allen Bateman:** Investigation; methodology; writing‐review and editing. **Amra Uzicanin:** Conceptualization; methodology; supervision; writing‐original draft; writing‐review and editing.

## CONFLICT OF INTEREST STATEMENT

The authors do not have any conflicts of interest to report.

## ETHICS STATEMENT

All components of ORCHARDS, including participant consent forms, were reviewed and approved by the Human Subjects Committees of the Education and Social/Behavioral Sciences IRB (initial approval on September 4, 2013; ID number: 2013‐1268) and the University of Wisconsin Health Sciences‐IRB (initial approval on December 5, 2013, with additional approvals as the protocol expanded and modified; ID number: 2013‐1357). The study is in full compliance with the Health Insurance Portability and Accountability Act of 1996 (HIPAA), Family Educational Rights and Privacy Act (FERPA), and all other federally mandated human subjects regulations. The US Office of Management and Budget approved all forms used in this study.

### PEER REVIEW

The peer review history for this article is available at https://www.webofscience.com/api/gateway/wos/peer-review/10.1111/irv.13171.

## Data Availability

Replication data are available at: https://dataverse.harvard.edu/dataset.xhtml?persistentId=doi:10.7910/DVN/GMDXLD.
